# Supplementary material for: COZOID: contact zone identifier for visual analysis of protein-protein interactions
Source: BMC Bioinformatics. 2018 Apr 6;19:125. doi: 10.1186/s12859-018-2113-6 (PMC5889581; doi:10.1186/s12859-018-2113-6)
Supplement: Supplementary file 2 — Software build. Executable binary file of the software tool. (ZIP 118,784 kb) [file 12859_2018_2113_MOESM2_ESM.zip › cozoid/java/docs/beansbinding-1.2.1-doc.zip!/org/jdesktop/observablecollections/class-use/ObservableList.html]

Uses of Interface org.jdesktop.observablecollections.ObservableList (Beans Binding)


---


|  |  |  |
| --- | --- | --- |
| **Overview** \| **Package** \| **Class** \| **Use** \| **Tree** \| **Deprecated** \| **Index** \| **Help** | |  |
| PREV   NEXT | **FRAMES**    **NO FRAMES**     **All Classes** |  |


---


## **Uses of Interface org.jdesktop.observablecollections.ObservableList**

| Packages that use ObservableList | |
| --- | --- |
| **org.jdesktop.observablecollections** | Provides support for observing changes to collection classes. |
| **org.jdesktop.swingbinding.impl** | Private implementation details for Swing binding. |

Uses of ObservableList in org.jdesktop.observablecollections

| Methods in org.jdesktop.observablecollections that return ObservableList | |
| --- | --- |
| `ObservableList<E>` | `ObservableCollections.ObservableListHelper.getObservableList()`             Returns the `ObservableList`. |
| `static <E> ObservableList<E>` | `ObservableCollections.observableList(java.util.List<E> list)`             Creates and returns an `ObservableList` wrapping the supplied `List`. |

| Methods in org.jdesktop.observablecollections with parameters of type ObservableList | |
| --- | --- |
| `void` | `ObservableListListener.listElementPropertyChanged(ObservableList list, int index)`             Notification than a property of an element in this list has changed. |
| `void` | `ObservableListListener.listElementReplaced(ObservableList list, int index, java.lang.Object oldElement)`             Notification that an element has been replaced by another in the list. |
| `void` | `ObservableListListener.listElementsAdded(ObservableList list, int index, int length)`             Notification that elements have been added to the list. |
| `void` | `ObservableListListener.listElementsRemoved(ObservableList list, int index, java.util.List oldElements)`             Notification that elements have been removed from the list. |

Uses of ObservableList in org.jdesktop.swingbinding.impl

| Methods in org.jdesktop.swingbinding.impl with parameters of type ObservableList | |
| --- | --- |
| `void` | `ListBindingManager.listElementPropertyChanged(ObservableList list, int index)` |
| `void` | `ListBindingManager.listElementReplaced(ObservableList list, int index, java.lang.Object oldElement)` |
| `void` | `ListBindingManager.listElementsAdded(ObservableList list, int index, int length)` |
| `void` | `ListBindingManager.listElementsRemoved(ObservableList list, int index, java.util.List elements)` |

---


|  |  |  |
| --- | --- | --- |
| **Overview** \| **Package** \| **Class** \| **Use** \| **Tree** \| **Deprecated** \| **Index** \| **Help** | |  |
| PREV   NEXT | **FRAMES**    **NO FRAMES**     **All Classes** |  |


---
